# Case Report: Paucisymptomatic College-Age Population as a Reservoir for Potentially Neutralization-Resistant Severe Acute Respiratory Syndrome Coronavirus 2 Variants

**DOI:** 10.4269/ajtmh.21-0542

**Published:** 2021-09-20

**Authors:** Benjamin W. Neuman, Wesley A. Brashear, Marcel Brun, Sankar P. Chaki, Rebecca S. B. Fischer, Sierra J. Guidry, Joshua E. Hill, Andrew E. Hillhouse, Charles D. Johnson, Melissa M. Kahl-McDonagh, Richard P. Metz, Allison C. Rice-Ficht, Jennifer A. Shuford, Tiffany A. Skaggs, Matthew A. Stull, David W. Threadgill, Yao Akpalu, Kurt Zuelke

**Affiliations:** ^1^ Global Health Research Complex, Division of Research, Texas A&M University, College Station, Texas;; ^2^ College of Science, College Station, Texas;; ^3^ Texas A&M Institute for Genome Sciences and Society, College Station, Texas;; ^4^ Texas A&M AgriLife Research, College Station, Texas;; ^5^ School of Public Health, Texas A&M University, College Station, Texas;; ^6^ College of Medicine, College Station, Texas;; ^7^ Texas Department of State Health Services, State Epidemiologist, Austin, Texas;; ^8^ Student Health Services, Texas A&M University, College Station, Texas;; ^9^ Brazos County Health Department, Epidemiology, College Station, Texas

## Abstract

To better understand the severe acute respiratory syndrome coronavirus 2 (SARS-CoV-2) variant lineage distribution in a college campus population, we carried out viral genome surveillance over a 7-week period from January to March 2021. Among the sequences were three novel viral variants: BV-1 with a B.1.1.7/20I genetic background and an additional spike mutation Q493R, associated with a mild but longer-than-usual COVID-19 case in a college-age person, BV-2 with a T478K mutation on a 20B genetic background, and BV-3, an apparent recombinant lineage. This work highlights the potential of an undervaccinated younger population as a reservoir for the spread and generation of novel variants. This also demonstrates the value of whole genome sequencing as a routine disease surveillance tool.

## CASE HISTORY

The patient, a college student in the 18–25 age demographic, presented for a COVID-19 screening test in early March 2021. During initial screening, the patient reported mild symptoms beginning 3 days earlier, did not seek medical care. Initial symptoms included a rare intermittent nonproductive cough, scratchy throat, and hoarseness.

The initial sample tested positive for severe acute respiratory syndrome coronavirus 2 (SARS-CoV-2) by nucleic acid amplification. A rapid antigen test performed 2 days later was also positive, and an oral swab polymerase chain reaction (PCR) from an oral swab collected 22 days after the initial positive sample were both positive. A follow-up saliva specimen collected 37 days after the initial positive sample was negative by PCR. Close contacts included a partner, three roommates, and work colleagues.

During the course of the illness, the patient also developed congestion, headache, laryngitis, mild hyposmia, and hypogeusia lasting only 1 day, and a brief fever of 101.2°F with chills 6 days after the initial positive test that resolved after about 2 hours and marked the end of the symptom cluster. The patient’s biggest complaints were laryngitis, lasting 26 days, with complete loss of voice for 3 days, and an overwhelming sense of “feeling off.” All symptoms were completely resolved 32 days after the initial positive sample, and 35 days after onset of COVID-19.

The patient denies any medical history of comorbidity, chronic condition, immune suppression, smoking, or asthma including childhood asthma, and had not received any COVID-19 vaccine. The patient had been in quarantine because of contact with two household members testing positive for SARS-CoV-2, last 5 days before the initial positive test, but did attend in-person activities during the 7 days prior to the initial screening, reporting consistent use of a face mask covering the nose and mouth when leaving home. Subsequent to infection, the patient reported that no close contacts tested positive or developed symptoms.

## VARIANT SEQUENCING

Ninety-three saliva samples from unique staff and student donors, collected from January through March 2021, were sequenced in this study. Sequencing was carried out by two methods: For whole genome SARS-CoV-2 sequencing, RNA was extracted from positive samples, and then sequencing was performed using one of two platforms: library preparation with the PerkinElmer Nextflex variant-seq SARS-CoV-2 kit, with sequencing using Illumina NovaSeq SP PE 2 × 150 flowcell v1.5 to generatean average of 8 million reads per sample, mapped and assembled using the Illumina DRAGEN-covid-pipeline-RUO-1.0.0; or, library preparation with the Swift SNAP SARS-CoV-2 amplicon panel, with sequencing using Illumina NextSeq to generate approximately 1 million reads per sample, mapped and assembled using the viralrecon pipeline.

Whole-genome sequencing of the initial sample revealed a novel variant B.1.1.7 genetic background, plus an additional mutation in the spike protein gene that would change glutamine 493 to arginine (Q493R). Mutations of Q493 resulting in an amino acid change to either arginine (R) or lysine (K) have been reported as an adaptation associated with resistance to monoclonal neutralizing antibodies in laboratory tests^1^ and Q493K confers resistance to the Regeneron antibody cocktail^2^ and bamlanivimab.^3^ It was recently reported that there are four major epitope classes on the SARS-CoV-2 spike protein receptor-binding domain where neutralizing antibodies bind, and that Q493R (BV-1) and E484K (not found in BV-1, but important in B.1.351 and B.1.617 variants) mediate roughly equivalent levels of resistance to only class-2 neutralizing antibodies, as defined in that study.^4^ Based on this, it is inferred that the Q493R mutation of BV-1 may confer resistance to some neutralizing antibodies, though we have not directly tested the neutralizing activity. We designated this novel variant BV-1, after the Brazos Valley public health district, uploaded to GISAID as hCOV-19/USA/TX-GHRC-BV1-EQ045269591/2021 (https://www.gisaid.org/). The BV-1 variant was reported to the relevant local, state, and national public health authorities.

## GENOMIC CONTEXT

**Figure 1.**
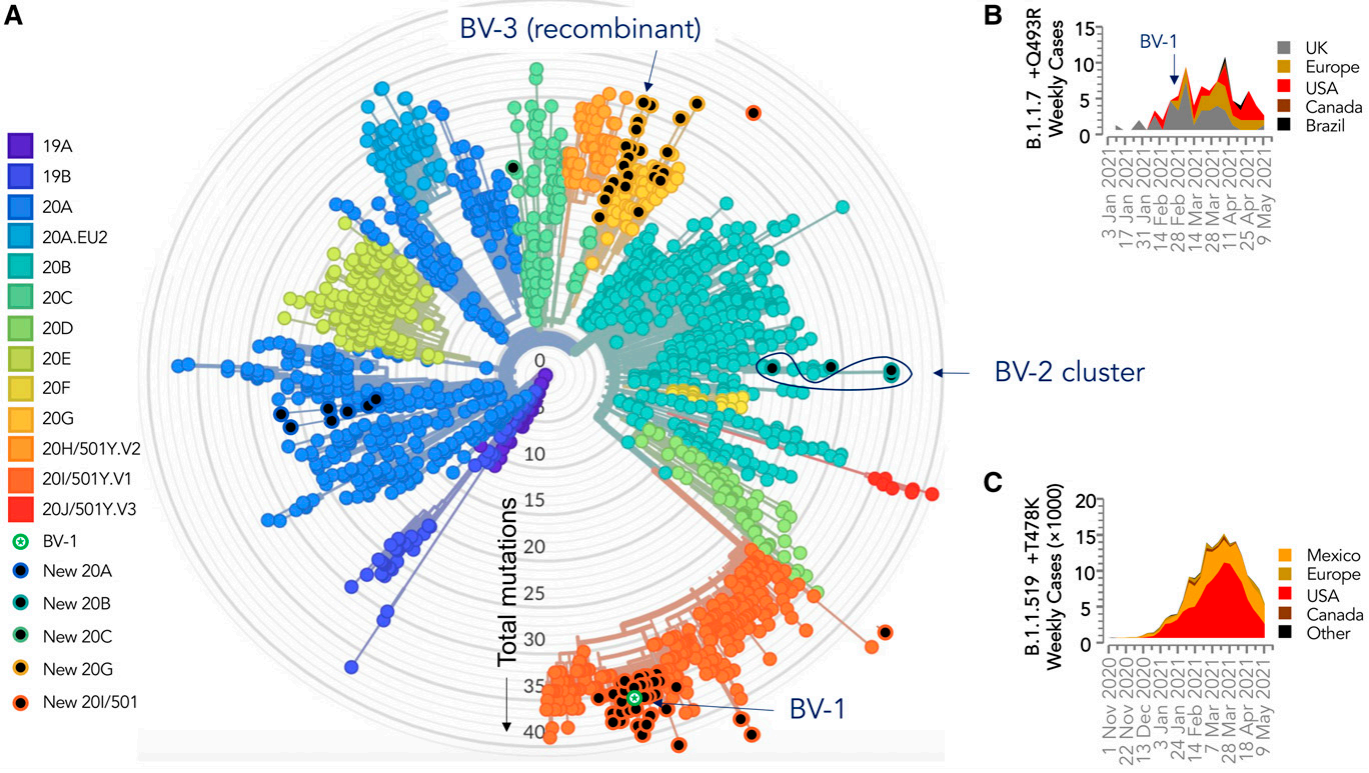
Phylogenetic placement of new severe acute respiratory syndrome coronavirus 2 (SARS-CoV-2) sequences within existing lineages. (**A**) Clade assignment and visual generated using the Nextstrain tool (clades.nextstrain.org), with distance from the center based on the number of mutations relative to the SARS-CoV-2/Wuhan-Hu-1 strain, accession number MN908947. New sequences reported in this study are shown as black dots, with variants designated BV-1, BV-2, and BV-3 indicated, with 4,000 other sequences from the Nextstrain globally subsampled daily dataset, accessed May 10, 2021. (**B**) Global reports of new genomes of BV-1-like viruses (B.1.1.7 + Q493R) per week, grouped by sampling date and location. (**C**) Global reports of new BV-2-like viruses (B.1.1.519 + T478K) per week.

The BV-1 genome was sequenced as part of the Texas A&M University COVID-19 surveillance program, which includes whole-genome sequencing of all positive samples. This sequence was part of a group of 93 genomes from samples tested in January through March 2021, and uploaded to GISAID (Figure 1). Of these, 56 (60%) sequences were of B.1.1.7/20I/501Y.V1 lineage; four isolated from early February through mid-March were of a B.1.1.519/20B lineage including three sequences with the T478K mutation that has been reported from the designated BV-2 (hCoV-19/USA/GHRC-BV2-EQ04518823/2021, hCOV-19/USA/GHRC-BV2-EQ04526485/2021 and hCoV-19/USA/GHRC-BV2-EQ04531246/2021), a lineage was first detected in the United States, but subsequently became common in Canada and Mexico with a peak near the end of March 2021; 22 were B.1.2/20G including BV-3, a genome very similar to other local 20G lineage sequences, except for the addition of a cluster of four spike mutations (P681H, T716I, S982A, and D1118H) that were not present in any local 20G lineage genomes, but were all present on all local 20I/501Y.V1 lineages, suggesting BV-3 arose from a recombination of a partial spike gene from a 20I/501Y.V1 strain on a 20G background (hCoV-19/USA/GHRC-BV3-EQ04527243/2021); five were B.1.596/20G; two were B.1.234/20A; two were B.1.243/20A; and one was B.1/20A (Figure 1).

At the time of writing, 109 BV-1-like genomes of B.1.1.7 lineage with a Q493R mutation have been reported to GISAID, with the earliest cases reported in the United Kingdom in January 2021, followed by appearances in the rest of Europe, Canada, Brazil, and 12 US states. The B.1.1.7/Q493R sequences form 12 clusters, each arising from a non-Q493R lineage, suggesting that Q493R is either more accessible or may have greater adaptive value on a B.1.1.7 background. BV-1 is genetically distinct from previously reported B.1.1.7/Q493R variants, forming a new cluster along with 45 other non-Q493R B.1.1.7 sequences, collected January 25 to March 11 from the same on-campus population. This suggests that BV-1 arose recently from the version of B.1.1.7 that was spreading locally.

## DISCUSSION

The viral sequence reported here has important implications for public health, owed to potential for increased transmissibility,^5^^,^^6^ combined with likely resistance to some neutralizing antibodies. Current data do not demonstrate widespread dissemination of BV-1 or other Q493R-containing variants at this time, but further monitoring may be warranted as increasing rates of vaccination change the competitive fitness landscape for SARS-CoV-2.

There is evidence that young adults transmit infection to older populations.^7^ This is concerning, from a public health perspective, because of the potential for new SARS-CoV-2 lineages to replicate and diversify in the younger population, which is at time of writing, relatively undervaccinated against COVID-19 compared with older populations. This report highlights the continued importance of measures to prevent viral transmission and the need for rigorous testing and genomic surveillance programs, including among young individuals with no symptoms or only mild illness.
